# Micro-porous PLGA/*β*-TCP/TPU scaffolds prepared by solvent-based 3D printing for bone tissue engineering purposes

**DOI:** 10.1093/rb/rbad084

**Published:** 2023-09-14

**Authors:** Luan P Hatt, Sylvie Wirth, Aapo Ristaniemi, Daniel J Ciric, Keith Thompson, David Eglin, Martin J Stoddart, Angela R Armiento

**Affiliations:** AO Research Institute Davos, 7270 Davos Platz, Switzerland; Institute for Biomechanics, ETH Zürich, 8093 Zürich, Switzerland; AO Research Institute Davos, 7270 Davos Platz, Switzerland; Institute for Biomechanics, ETH Zürich, 8093 Zürich, Switzerland; AO Research Institute Davos, 7270 Davos Platz, Switzerland; AO Research Institute Davos, 7270 Davos Platz, Switzerland; AO Research Institute Davos, 7270 Davos Platz, Switzerland; UCB Pharma, SL1 3WE Slough, UK; AO Research Institute Davos, 7270 Davos Platz, Switzerland; Mines Saint-Étienne, Université de Lyon, Université Jean Monnet, INSERM, U1059, 42023 Sainbiose, Saint-Étienne, France; AO Research Institute Davos, 7270 Davos Platz, Switzerland; Medical Center, Faculty of Medicine, Albert-Ludwigs-University of Freiburg, 79106 Freiburg, Germany; AO Research Institute Davos, 7270 Davos Platz, Switzerland; UCB Pharma, SL1 3WE Slough, UK

**Keywords:** solvent-based printing, porosity, regenerative scaffold, osteogenesis, mesenchymal stromal cells

## Abstract

The 3D printing process of fused deposition modelling is an attractive fabrication approach to create tissue-engineered bone substitutes to regenerate large mandibular bone defects, but often lacks desired surface porosity for enhanced protein adsorption and cell adhesion. Solvent-based printing leads to the spontaneous formation of micropores on the scaffold’s surface upon solvent removal, without the need for further post processing. Our aim is to create and characterize porous scaffolds using a new formulation composed of mechanically stable poly(lactic-co-glycol acid) and osteoconductive β-tricalcium phosphate with and without the addition of elastic thermoplastic polyurethane prepared by solvent-based 3D-printing technique. Large-scale regenerative scaffolds can be 3D-printed with adequate fidelity and show porosity at multiple levels analysed via micro-computer tomography, scanning electron microscopy and N_2_ sorption. Superior mechanical properties compared to a commercially available calcium phosphate ink are demonstrated in compression and screw pull out tests. Biological assessments including cell activity assay and live-dead staining prove the scaffold’s cytocompatibility. Osteoconductive properties are demonstrated by performing an osteogenic differentiation assay with primary human bone marrow mesenchymal stromal cells. We propose a versatile fabrication process to create porous 3D-printed scaffolds with adequate mechanical stability and osteoconductivity, both important characteristics for segmental mandibular bone reconstruction.

## Graphical abstract

**Figure:**
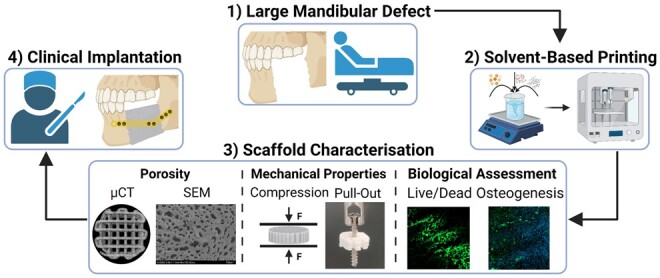

## Introduction

Trauma, tumour, infection and congenital malformation are among the main causes of large segmental mandibular defects, which require a complex surgical procedure for successful functional and aesthetic reconstruction [1]. The current standard of care (SOC) is autologous bone grafting from either the iliac crest or the fibula [2], which is associated with several drawbacks such as limited tissue availability, donor site morbidity, lack of patient-specific graft geometry and excessive resorption. To improve patient care, an effective alternative is required ideally overcoming the aforementioned disadvantages. The replacement of the current SOC should be a product that not only possesses mechanical stability enabling fixation with a plate-screw system and biological properties such as biocompatibility, biodegradability and osteoconductivity, but it should also provide practical advantages such as simple, cost-effective and versatile biofabrication process.

3D printing is a powerful fabrication tool enabling the creation of patient-specific and mechanically stable scaffolds in a cost- and time-effective manner. A very common printing approach is fused deposition modelling (FDM) that involves heat-melting and deposition of thermoplastic polymers, such as polycaprolactone (PCL) or polylactic acid (PLA) [3]. However, they degrade at temperatures higher than 200°C [4] and have been shown to undergo a 48% reduction of PLA average molecular weight after printing at 186°C [5]. This degradation is detrimental for the performance of the 3D-printed scaffold [6, 7]. Additionally, FDM printing requires the use of a filament, which makes the combination of osteoconductive calcium phosphate (CaP) material or other compounds tedious and requires a pre-processing step involving melt-blending extruding to create a filament composed of the desired mixed composition [8–12]. Finally, FDM printing of PLA is associated with the formation of an undesirable smooth surface topography [13, 14]. Surface microroughness and porosity are both important features for protein adsorption, cell adhesion and tissue infiltration for enhanced osteogenesis [15–17]. A variety of postprocessing surface modification strategies can be applied to improve these features such as plasma treatment, coating or protein grafting [18–20]. An elegant printing alternative to FDM is low-temperature solvent-based printing. Ink components are easily mixed, avoiding extreme temperatures and consequent degradation, to create a 3D printable blend. The post-printing removal of the solvent spontaneously leads to the creation of micropores on the surface of the scaffold, beneficial for cell adhesion and osteogenesis [16] without the need of further chemical surface modifications [21, 22].

Our aim is to fabricate a micro-porous scaffold prepared by a newly developed solvent-based 3D-printing technique for bone tissue engineering purposes. To replace commonly used toxic organic solvents, we used water-soluble and non-toxic ethylene carbonate (EC). To demonstrate the versatility of the method, we modulated material properties by creating a composite made of poly(lactic-co-glycol acid) (PLGA), for printability and mechanical strength, β-tricalcium phosphate (β-TCP) for osteoconductivity, with and without the addition of thermoplastic polyurethane (TPU), which is known to be biodegradable [23, 24] and elastic [25], and subsequently characterized the material properties. Increasing the elastic properties of biomaterials can be beneficial for the mandibular bone repair regarding the mandible’s chewing function. As a control, the newly developed inks were compared with a commercially available 3D printable CaP ceramic, OsteoInk^®^. In this study, we report an advanced low-temperature solvent-based printing approach to develop a versatile, medically relevant manufacturing process that allows fabrication of patient-specific regenerative bone substitutes for bone defect repair.

## Materials and methods

**Figure 1.**
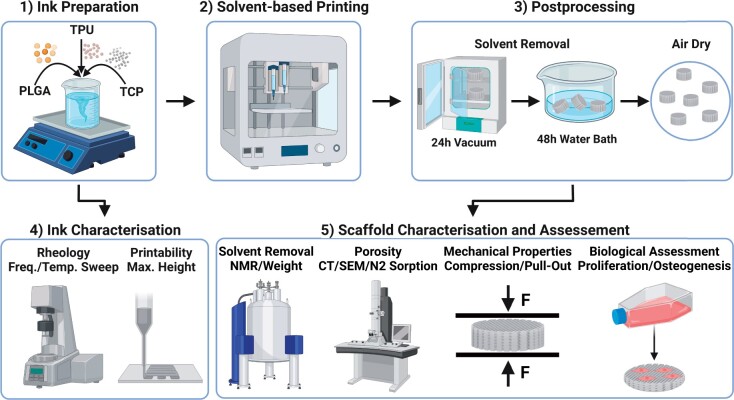
Overview of the methods: (1) ink preparation, (2) solvent-based printing, (3) scaffold postprocessing, (4) ink characterization and (5) scaffold characterization and assessment. Created with BioRender.com.

Human bone marrow aspirates are obtained with informed consent of all donors and with full approval from the Ethics Committee of the University of Freiburg Medical Centre (EK-Freiburg: 135/14, 25 March 2014) and the ethical commission of Zürich (KEK-ZH-NR: 2016-00141). All reagents are purchased from Sigma-Aldrich unless otherwise stated. An overview of the methods is reported in Figure 1.

### Preparation of PLGA/β-TCP ± TPU ink

TPU (ROLASERIT^®^, AM Polymers GmbH) powder is dissolved in EC at 120°C using a hot plate stirrer and a rotor mixing system. After cooling the hot plate stirrer to 90°C, PLGA (PURASORB^®^ PLG 8531, 85/15 L-lactic/Glycolide, Corbion, Amsterdam, Netherlands) powder is added and mixed until dissolution. Subsequently, the system is cooled to 80°C, β-TCP powder (BABI-TCP-N100, Berkeley Advanced Biomaterials) is added, and the blend stirred overnight using a Hei-TORQUE Expert 200 (Heidolph Instruments) set to 30 rpm to obtain a viscous, homogenous ink. The ink is transferred to 3 cc syringe barrels (Nordson EFD) and kept at −20°C until printing. Two ink formulations, PLGA/β-TCP − TPU (without TPU) denoted as group 1 (G1) and PLGA/β-TCP + TPU (addition of TPU) denoted as group 2 (G2), are prepared and the ratio between individual components is presented in Table 1.

**Table 1.** Ink formulations

Group	PLGA (w/V)	β-TCP (w/V)	TPU (w/V)
1	40%	20%	—
2	40%	20%	10%

### Rheological characterization of the inks

Viscoelastic properties of the inks are investigated using an Anton Paar MCR-302 rheometer (Anton Paar). Oscillatory and rotational tests are performed using a flat-plate geometry with the gap set at 0.5 mm. An amplitude sweep test is carried out with strain ranging between 0.01% and 100% at constant angular velocity of 10 rad/s and a temperature of 80°C. Viscosity is evaluated using a frequency sweep test (*N* = 3) between 0.1 and 100 Hz, with a constant strain of 1% based on the amplitude test and a temperature of 80°C. A temperature sweep test (*N* = 3) is performed with the temperature ranging from 80°C to 40°C to investigate the rheological properties of the thermo-responsive ink with constant strain of 0.1% and frequency of 1 Hz. Storage modulus (*G*′) and loss modulus (*G*″) values are acquired for all tests.

### Design of 3D-printed scaffolds

Two different scaffold structures are used in this study and designed using BioCAD software (RegenHU). Design 1 is round and has a porous grid structure. Design 2 is a round dense disc with only two layers and can be press-fit into 24-well-plates, especially practical for cell seeding. Using the discs (Design 2) for cell experiments allows for higher standardization of cell seeding and attachment on top of the biomaterial compared to the Design 1, which can lead to uneven cell-attachment starting point between groups. Scaffold dimensions and corresponding measurement are summarized in Table 2.

**Table 2.** Characteristics of 3D-printed scaffold structures and physico-chemical characterizations performed

Design	Schematic	Dimensions (mm)	Measurement/characterization
1	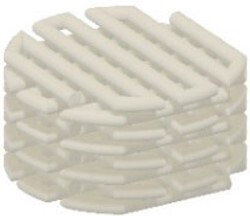	D = 5 H = 4 LS = 0.4	NMR Weight solvent extraction test (*N* = 3) Degradation/swelling (*N* = 6) µCT scanning (*N* = 3) SEM images N_2_ sorption Mechanical compression test (*N* = 13/14) Screw pull-out test (*N* = 12) Indirect cytotoxicity test (*N* = 4)
2	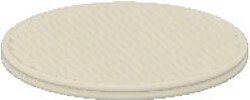	D1 = 7.5 D2 = 13 H = 0.8 LS = 0.5 OS = 0.4	DNA quantification (*N* = 3) Live/dead staining Alizarin red staining (*N* = 3) Osteogenesis (*N *= 2)

D, diameter; H, height; LS, line spacing; D1, diameter for 96-well-plate; D2, diameter for 48-well-plate; OS, offset; NMR, nuclear magnetic resonance; µCT, micro-computed tomography; SEM, scanning electron microscope; N_2_, nitrogen.

### Solvent-based 3D printing and postprocessing of PLGA/TCP ± TPU and Osteoink^®^

#### Scaffold fabrication

Low-temperature solvent-based 3D printing is performed using 3D Discovery^®^ bioprinter (RegenHU). Ink-laden cartridges are equipped with a stainless steel needle with a 0.5-mm inner diameter and a piston. During printing the cartridge heater is set at 80°C and a pressure between 0.5 and 1.8 bars is applied for the different groups. The velocity of the print head is 4 mm/s, and the layer height is set at 0.4 mm. The ink is extruded onto a dry glass slide which is mounted onto an aluminium cool plate with the temperature kept between 3°C and 10°C. The printing of the commercially available Osteoink^®^ (RegenHu) is performed at room temperature onto a glass slide using the same needle size, pressure, print head velocity and layer height.

#### Postprocessing

To remove the solvent, the 3D-printed PLGA/β − TCP ± (TPU) scaffolds are placed first in a vacuum oven at room temperature for 24 h and then in a water bath at 37°C for 48 h (4× Milli-Q^®^ water changes). Scaffolds are then air dried and stored at room temperature until further use. 3D-printed discs (Design 2) are size-fitted for a 96-well-plate and 48-well-plate using a hollow punch with a dimension of 7 mm and 11 mm in diameter, respectively. The discs are sterilized using a cold ethylene oxide gas protocol, degassed under vacuum for at least 5 days, and press-fit into the cell-culture wells under sterile conditions prior to use. 3D-printed Osteoink^®^ scaffolds are hardened post-printing using a steam autoclave (Belimed Infection Control, Belimed Sauter AG) as previously described [26].

#### Post-printing solvent removal confirmation

To verify complete removal of the solvent, the 3D-printed scaffolds (Design 1) are dried and weighed during the extraction process after 0, 24 and 48 h (*N* = 3). 1H nuclear magnetic resonance (NMR) is carried out to further confirm complete solvent removal using Bruker Avance AV-500 18 MHz NMR spectrometer. Prior to measurement, 10–30 mg/ml of scaffolds are dissolved in trichloromethane/chloroform (Carl Roth GmbH) for 3 h on a spinning wheel, centrifuged and the supernatants transferred into NMR tubes. The presence of EC is defined by the peak at 4.541 ppm of the four hydrogen protons.

### Micro-computed tomography

Visualization of 3D-printed scaffolds is performed via micro-computed tomography (µCT) using vivaCT 40 (SCANCO Medical AG) with 45 kVp voltage, 177 μA current and 10.5 μm isotropic voxel size. Printing porosity of 3D-printed scaffolds is calculated using the Amira image processing software (V. 6.4.0; Thermo Fisher Scientific, Waltham, MA) (*N* = 3). A standardized image processing protocol is used. Firstly, the scaffold is segmented and using a filling algorithm, the inside of the segmentation is defined, leaving the total volume of the scaffold and the inside segmented. The scaffold is segmented again and combined with the total volume mask. With the combined mask, the scaffold’s porosity is calculated as a percentage of the total volume divided by the volume of the scaffold mask.

### Scanning electron microscopy

Surface topography of 3D-printed scaffolds (Design 1) is visualized using a scanning electron microscope (SEM) (Hitachi SU 5000). Upon solvent extraction, scaffolds are mounted onto stubs using carbon conductive cement Leit-C, dried overnight and subsequently sputter coated with a layer of platinum/palladium using CCU-010 Metal Sputter Coater (Safematic). SEM images are taken at 3 kV voltage, 10 mA beam current and a working distance of 5 and 50 mm for surface images and overview images, respectively. Quantification of surface porosity is based on the close-up surface images and obtained using ImageJ software (*N* = 7).

### N_2_ sorption

Mesoporous properties (2–50 nm) 24 of 3D-printed scaffolds (Design 1) are determined via nitrogen (N_2_) sorption using Micromeritics ASAP 2020 (Instrument Corp.) under liquid N_2_ (−195.8°C). Scaffolds (ca. 0.5 g) are degassed at room temperature under vacuum for 48 h prior to measurement. Specific surface area (SSA) and desorption average pore width (4 V/A) values are determined by applying the Brunauer–Emmett–Teller (BET) theory.

### Degradation/swelling test

To investigate degradation and swelling, Design 1 3D-printed scaffolds (N = 6) are placed individually in a well of a 6-well-plate and incubated in 5 ml phosphate-buffered saline (PBS) for 28 days at 37°C under constant shaking. Beforehand, the weight of the scaffolds is measured in dry state, which is used as the normalizer. Wet scaffolds are measured at day 1, 7, 14 and 28. After the incubation period, the scaffolds are air dried for 2 days and the weight is measured in dry state. The pH of the PBS is measured at day 1, 7, 14 and 28 to identify possible reduction caused by scaffold degradation.

### Compression test

Mechanical compression tests (Design 1, G1 and G2: *N* = 14, Osteoink: *N* = 13) of pre-wetted 3D-printed scaffolds are performed using Instron 5866 (Instron) equipped with a 1-kN load cell. The compression test is divided into two parts: (i) four loading-unloading cycles at the rate of 1%/s to 15 or 8% compressive strain are applied to the PLGA/TCP ± TPU and the Osteoink^®^ scaffolds respectively, and the slope from the linear region of the stress–strain curve of the fourth cycle is taken to calculate the Young’s modulus; (ii) subsequent ultimate test using a speed of 0.1%/s until a strain of 50% to break the scaffold and measure yield strain, yield stress and toughness at yield.

### Screw pull-out test

Screw pull out tests of 3D-printed scaffolds (Design 1) are performed using Instron 5866 equipped with a 50-N load cell (*N* = 12). Prewetted scaffolds are divided into two groups: (i) no pre-drilling is applied; (ii) scaffolds are pre-drilled using 1.8 mm drill head (DePuy Synthes). An 18 mm ‘MatrixMANDIBLE’ screw (DePuy Synthes) with diameter of 2 mm is screwed into the scaffolds. The screw is installed in a custom-made screw holder that is connected to the load cell, placed into a chamber with a slot and pulled out with a velocity of 0.25 mm/s and the maximum force during testing is recorded.

### Cell culture of L929 fibroblasts

L929 fibroblasts (85011425, mouse C3H/An connective tissue, Sigma) are cryopreserved according to the company’s instructions in liquid nitrogen. Upon thawing, 1 × 10^6^ cells are seeded in a T300 flask (cell density 3.33 × 10^3^ cells/cm^2^) for culture expansion. The expansion medium (EM) consists of low glucose (1 g/l)-DMEM (LG-DMEM) (Gibco, Carlsbad) supplemented with 10% (v/v) foetal bovine serum (Corning) and 100 U/ml penicillin, 100 µg/ml streptomycin (PEN/STREP) (Gibco). Cells are cultured under standard cell culture conditions of 37°C with 5% CO_2_ and 90% humidity with three media changes per week.

### Cell isolation and culture of human bone marrow-derived mesenchymal stromal cells

Isolation and cryopreservation of human bone marrow mesenchymal stromal cells (hBM-MSCs) from bone marrow aspirates is performed as described previously [27], as well as cell culture expansion as described previously [28]. hBM-MSC donor details are as follows: Donor A, 52-year old female, spine vertebral body aspirate, Donor B, 51-year old female, spine vertebral body aspirate; Donor C, 48-year old female, spine vertebral body aspirate; Donor D, 74-year old female, spine vertebral body aspirate; Donor E, 44-year old male, iliac crest cancellous bone.

### Indirect cytotoxicity test via CellTiter-Blue^®^

The indirect toxicity test of 3D-printed scaffolds (Design 1) is carried out according to the ISO 10993 guideline using the L929 fibroblasts (*N* = 4). Cells are harvested using 0.05% Trypsin-EDTA (Gibco) and seeded on 96-well-plates at a density of 1 × 10^4^ cells/well in sic replicates. For the first 24 h, cells are cultured in EM. In the meantime, 3D-printed scaffolds are incubated in EM in triplicates for 24 h to obtain conditioned media (CM1-3). At this point (day 0), cells are switched into their corresponding culture media: (i) positive control: EM supplemented with 0.1% Triton X-100; (ii) negative control: EM; (iii) CM1-3. CellTiter-Blue^®^ (CTB) is performed at day 1 and day 3. Media are removed and EM supplemented with 16.6% (v/v) CTB reagent is added. After 4 h of incubation at 37°C with 5% CO_2_ and 90% humidity, the supernatant is transferred into a 96 clear bottom well plate (Corning, New York, USA) and fluorescence is read at 560/590 nm using Infinite^®^ 200 PRO plate reader (Tecan Trading AG).

### DNA quantification and live/dead staining on 3D-printed discs

hBM-MSCs are harvested using 0.05% Trypsin-EDTA and seeded at a density of 8.75 × 10^3^ cells/well (10 × 10^3^ cells/cm^2^) in duplicates on either tissue culture plastic as control or onto 3D-printed discs (Design 2) and cultured in EM. At day 1 and day 7, DNA quantification is performed using the CyQuant™ Cell Proliferation Assay (Invitrogen) according to the manufacturer’s instructions (*N* = 3). In short, cells are lysed with 0.1% Triton X-100 in 10 mM TrisHCl for 2 h at 4°C, cell lysate is transferred into a 96 clear bottom well plate, working solution containing dye is added and fluorescence is read at 490/530 nm using Infinite^®^ 200 PRO plate reader.

At day 1 and day 7, cells are washed with PBS, stained with staining solution that contains 1 µM Calcein, AM (Invitrogen) and 1 µM Ethidium Homodimer-1 (Invitrogen) in serum-free LG-DMEM and incubated at 37°C for 1 h. The staining solution is rinsed with PBS and the cells are subsequently imaged using a confocal microscope (LSM800, Leica Microsystems).

### Alizarin red staining and quantification of 3D-printed discs

3D-printed discs (Design 2) are stained with Alizarin red (ScienCell Research Laboratories) according to the manufacturer’s instructions (*N* = 3). In short, the discs are incubated with 40 mM Alizarin red solution for 1 h at room temperature under gentle movement. The discs are washed with Milli-Q^®^ water until no discoloration is visible. The stained discs are imaged and incubated with 10% (v/v) acetic acid for 30 min at room temperature on an orbital shaker to extract the dye. The solution is transferred into Eppendorf tubes (Eppendorf, Hamburg, Germany), incubated for 10 min at 85°C, cooled on ice for 5 min and centrifuged at 20 000 × g for 15 min at room temperature. 10% (v/v) ammonium hydroxide is used to adjust the pH to 4.1–4.5 of the transferred supernatant. The absorbance at 405 nm is measured using Infinite^®^ 200 PRO plate reader.

### Osteogenic assessment of hBM-MSCs cultured on G1(−TPU) osteogenic differentiation

hBM-MSCs are harvested using 0.05% Trypsin-EDTA and seeded at a density of 28.5 × 10^3^ cells/well (15 × 10^3^ cells/cm^2^) in duplicates onto either coverslips (SARSTEDT AG) cultured in osteocontrol medium (EM) or 3D-printed discs of G1(−TPU) (Design 2) and cultured either in osteocontrol—or osteogenic medium (EM supplemented with 10 nM dexamethasone, 5 mM β-glycerophosphate and 50 µg/ml L-ascorbic acid-2-phosphate) for 28 days under normal culture conditions (*N* = 2).

### Quantification of alkaline phosphatase activity and DNA content

At day 14, alkaline phosphatase (ALP) activity is measured as described previously [28]. In short, after cell lysis with 0.1% Triton X-100 in 10 mM TrisHCl, the enzymatic reaction is started by adding alkaline buffer solution, substrate solution (25 mg/ml phosphate substrate in 1 mM diethanolamine) and Milli-Q^®^ water and stopped by adding 0.1 M NaOH solution after 15 min at 37°C. The absorbance is read at 405 nm using the Infinite^®^ 200 PRO plate reader. ALP activity is normalized to the DNA content, which is performed as described in the previous section (DNA Quantification and Live/Dead Staining on 3D-printed Discs).

### Staining of mineral deposition

At day 28, cells are fixed with 10% neutral buffered formalin for 30 min, permeabilized with 0.25% Triton X-100 in PBS for 20 min and stained with 2 µg/ml 4′,6-Diamidino-2-phenylindole solution for 10 min with a PBS wash before each step. Mineral deposition is stained with OsteoImage™ Mineralization Assay (Lonza) according to the manufacturer’s instructions and imaging is performed using a confocal microscope (LSM800, Leica Microsystems).

### RNA isolation and RT-qPCR

Cells are harvested for gene expression analysis, RNA isolation at day 28 and real-time quantitative PCR is performed using the QuantStudio™ Flex Real-Time PCR System as described previously [28]. Reverse transcription is performed using the Superscript Vilo cDNA Synthesis Kit (Thermo Fisher Scientific) according to the company’s instructions. Gene expressions of osteo-relevant markers: *ALPL* (encodes for ALP), *IBSP* (encodes for bone sialoprotein), *SP7* (encodes Osterix) and *SPP1* (encodes Osteopontin) are investigated. Primer sequences used are listed in Table 3. The ΔΔCt method is applied for data analysis using RPLP0 as an endogenous normalizer and day 0 samples as a calibrator.

**Table 3.** Primers/probes used for qPCR

Gene		**Assay on demand** ^a^	
*ALPL*		*Hs00758162_m1*	
*IBSP*		*Hs00173720_m1*	
*SP7*		*Hs00541729_m1*	
*SPP1*		*Hs00959010_m1*	
**Gene**	**Forward**	**Reverse**	**Probe**
*RPLP0*	*5'-TGG GCA AGA ACA CCA TGA TG-3'*	*5'-CGG ATA TGA GGC AGC AGT TTC-3'*	*5'-AGG GCA CCT GGA AAA CAA CCC AGC-3'*

*ALPL*, alkaline phosphatase, biomineralization associated; *IBSP*, integrin binding sialoprotein; *SP7*, Sp7 transcription factor; *RPLP0*, ribosomal protein lateral stalk subunit P0; *SPP1*, osteopontin, organic component of bone matrix.

a TaqMan^®^ Gene Expression Assay (Applied Biosystems^®^).

### 3D printing of a patient-specific mandible defect-sized PLGA/β-TCP scaffold

The self-created defect in the mandible SYNBONE^®^ is µCT-scanned using the vivaCT 40. The defect geometry is exported as a STL. file using the Amira image processing software and converted into the g-code using the MM Converter software (RegenHu). Solvent-based printing is applied to the PLGA/β-TCP (G1(−TPU)) ink to create a patient-specific mandible defect-sized scaffold to demonstrate the precise printability of relevant defect sizes.

### Statistics

Statistical analysis is performed using GraphPad Prism software version 9.3.1 (GraphPad Software). An unpaired parametric test based on the Welch’s test, which assumes different standard deviation is applied to the data of the printing porosity, surface porosity, gene expression and Alizarin red quantification. A one-way ANOVA is applied to the data of the Young’s modulus, yield stress—and strain. A two-way ANOVA is applied to the data of the peak displacement, screw pull-out test, DNA quantification and ALP activity. *P*-values higher than 0.05 are not significant and marked as ns.

## Results

### Both PLGA/β-TCP ± TPU inks demonstrate adequate rheological properties for 3D printing and superior printability compared to OsteoInk^®^

**Figure 2.**
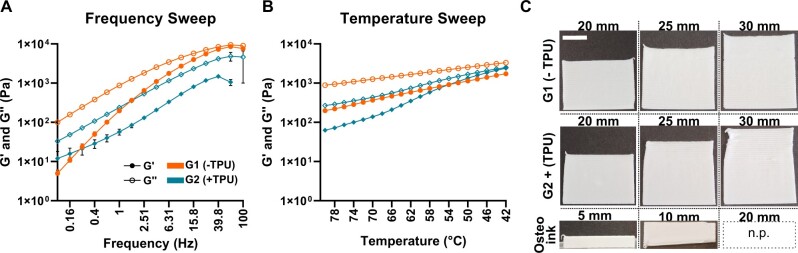
Ink rheology and maximum printability: rRepresentative sweep curves (*N* = 3) of (**A**) frequency and (**B**) temperature; (**C**) three different inks (G1, G2 and OsteoInk^®^) are printed to the maximal height until artifacts or failure (collapsed structure for Osteoink at 10 mm, top view); scale bar: 1 cm, n.p. = not possible.

In the frequency sweep test, both inks remain viscous (*G*′ < *G*″) with increasing frequency when the temperature and the amplitude is kept constant at 80°C and 1%, respectively (Figure 2A). *G*′ values start at 5 ± 1.1 Pa and 12 ± 6 Pa and increase up to 9433 ± 887 Pa and 1480 ± 10^4^ Pa for G1(−TPU) and G2(+TPU), respectively. The addition of TPU decreases both the storage and loss modulus at frequencies higher than 0.25 Hz. Temperature-dependent viscosity changes are measured using a temperature sweep test (Figure 2B). Viscous behaviour (*G*′ < G″) dominates for G1(−TPU) ink with decreasing temperature, while for G2(+TPU) ink solid-like behaviour already starts to occur at 42°C. *G*′ values start at 198 ± 1.1 Pa and 63 ± 6.4 Pa and increase up to ca. 1736 ± 10^2^ Pa and 2511 ± 252 Pa for G1(−TPU) and G2(+TPU), respectively.

The addition of TPU accelerates solidification of the ink with decreasing temperatures (Figure 2B). Rheologic characteristics of both inks display viscous behaviour at printing temperatures, which is important for successful printing.

Printability is measured by investigating the maximum printing height until failure or artifact (Figure 2C). Single line printing of G1(−TPU) ink can be achieved up to 30 mm without visual artifacts, while with G2(+TPU) ink artifacts appear after a height of 25 mm. OsteoInk^®^ shows a successful single line printing up to 5 mm, but construct collapse occurs when a 10-mm height is attempted. Printing the OsteoInk^®^ up to 20 mm is therefore not possible. Both G1 and G2 inks show artifact-free printing for constructs larger than 25 mm, while the OsteoInk^®^ collapses after 5 mm of printing.

### Total solvent removal is confirmed and does not jeopardize filament shape

**Figure 3.**
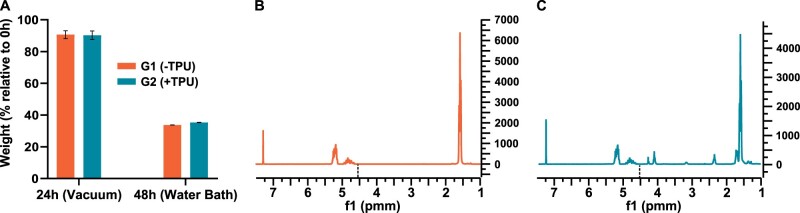
Solvent removal: (**A**) scaffold weight relative to 0 h after incubation under vacuum at 24 h and water bath at 48 h (*N* = 3); (**B**) representative NMR spectrum of G1(−TPU) and (**C**) representative NMR spectrum of G2(+TPU). The NMR peak of EC is marked with a black dotted line in both spectra at 4.45 pmm.

The weight of the 3D-printed scaffold is measured during the two-step solvent removal process, which starts with a 24-h step under vacuum and ends at 48-h following an additional 24-h step in a water bath (Figure 3A). The vacuum step causes a reduction of ca. 10% of the scaffold weight, while the water step accounts for additional ca. 55% weight lost in both groups, which corresponds to the ca. 65% weight ratio of the solvent within the scaffold before removal. Total solvent removal is indirectly confirmed by 1H NMR after the water step at 48 h. No EC peak is present at 4.54 ppm in both spectra of G1(−TPU) (Figure 3B) and G2(+TPU) (Figure 3C).

**Figure 4.**
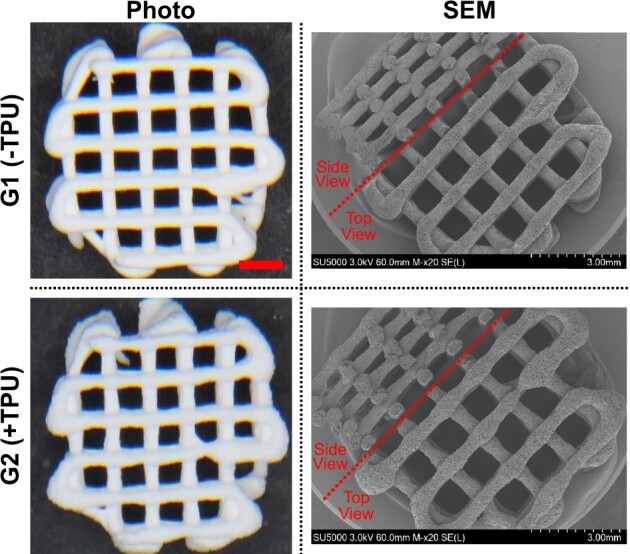
Representative images of 3D-printed scaffold (Design 1): photo SEM; scale bar = 1.5 mm.

Representative photo—and SEM images of 3D-printed scaffolds (Design 1) (Figure 4) after solvent removal illustrates the shape of the overall scaffold, the filament and the surface, as well as the side view of both groups. Filament shape remains intact after solvent removal.

**Figure 5.**
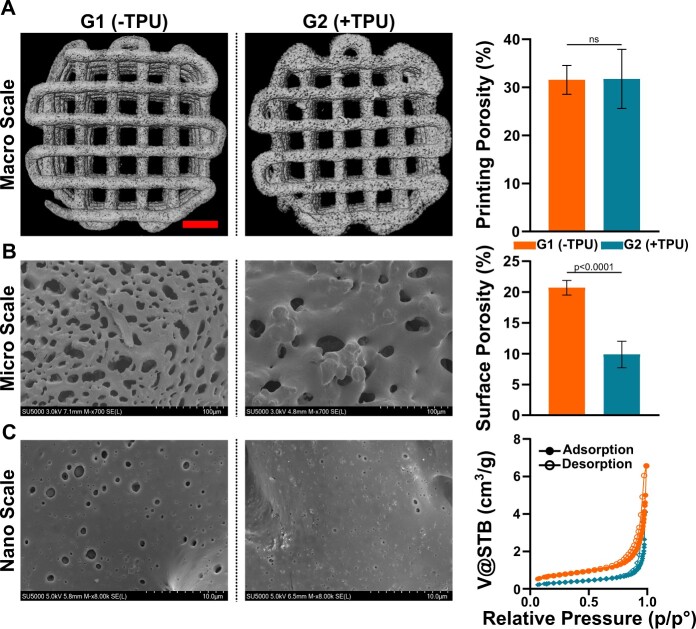
Three-level porosity measurement from macro to nano through micro scale: (**A**) printing porosity (100 µm–1 mm) based on µCT (*N* = 3), scale bar = 1.5 mm; (**B**) surface porosity (2–50 µm) (*N* = 7) based on SEM and (**C**) nano- and mesoporosity based on nitrogen sorption isotherms (2–100 nm) of 3D-printed scaffolds. ns, not significant.

Increased surface roughness is visible in G2(+TPU) compared to G1(−TPU) as noticeable from µCT images (Figure 5A). The side view of cut filaments in the SEM images show slightly elliptic shape in both groups.

### Porosity is present at three levels from macro to micro to nano scale

The macro-scale printing porosity, representing the negative space within the 3D-printed scaffold in-between the struts, is calculated to be 31.5 ± 3.0% for G1(−TPU) and 32 ± 6.1% for G2(+TPU) (Figure 5A). No statistical differences are observed between the two groups indicating indistinguishable shape fidelity. Micro-scale SEM images of G1(−TPU) show smaller, but a higher number of pores compared to G2(+TPU) (Figure 5B). The SEM images confirms increased topography roughness of the second group, which was already visible on µCT images. G1(−TPU) has a surface porosity of 21 ± 1.2%, a significantly increased number compared to 10 ± 2.2% of G2(+TPU). Nano-scale porosity is visible in the SEM images for both groups (Figure 5C). The adsorption and desorption isotherm profiles of both groups are considered type 3 isotherms and show H3 type hysteresis loops according to the IUPAC classification [29]. The addition of TPU decreases mesoporosity, the SSA, while the desorption average pore width value is increased (Table 4). Solvent-based printing allows for a three-level macro, micro and nano-scale porosity, the microscale decreases when TPU is added, while the macro and nanoscale remain unchanged.

**Table 4.** Specific surface area and desorption average pore width values (4 V/A by BET) of 3D-printed scaffolds after solvent removal

Value	**G1(**−**TPU)**	G2(+TPU)
SSA (m^2^/g)	2.5 ± 0.05	1.2 ± 0.02
Desorption average pore width (nm)	15	19

SSA, specific surface area.

### 3D-printed scaffolds swell without acidic change in pH

**Figure 6.**
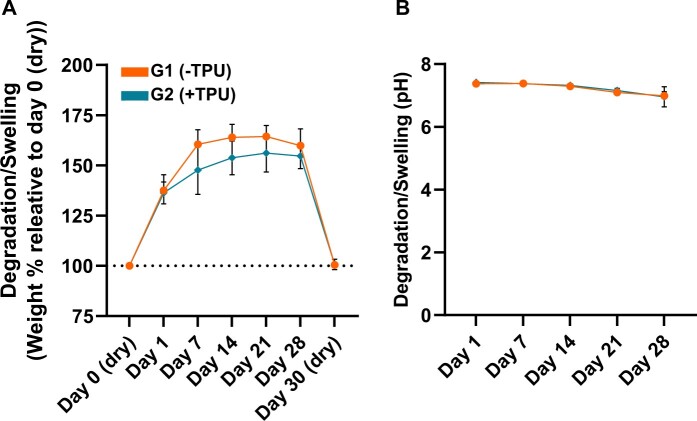
Degradation/swelling of 3D-printed scaffolds (Design 1) incubated in PBS at 37°C measured at day 1, 7, 14, 21 and 28 (*N* = 6): (**A**) weight percentage relative to day 0 (dry) and (**B**) pH of PBS.

3D-printed scaffolds (Design 1) swell upon incubation in PBS at 37°C up to 164.4% ± 5.5 in G1(−TPU) and 156.1% ± 9.8 in G2(+TPU) relative to day 0 (dry) until 21 days, after which the weight of the scaffolds slightly decreases at day 28 (Figure 6A). The majority of the swelling occurs in the first 7 days. The addition of TPU reduces the swelling effect. The weights of the dry scaffolds after 28 days of incubation is measured at 100.4% ± 0.1 in G1 and 100.7% ± 2.6 in G2 indicating that the scaffolds did not degrade in PBS under *in vitro* conditions. The pH of the PBS slightly decreases over the incubation period but remains close to neutral between ca. 7.4 and 7 in both groups confirming no acidic change of the milieus (Figure 6B).

### The addition of TPU leads to a decreased compressive stiffness

**Figure 7.**
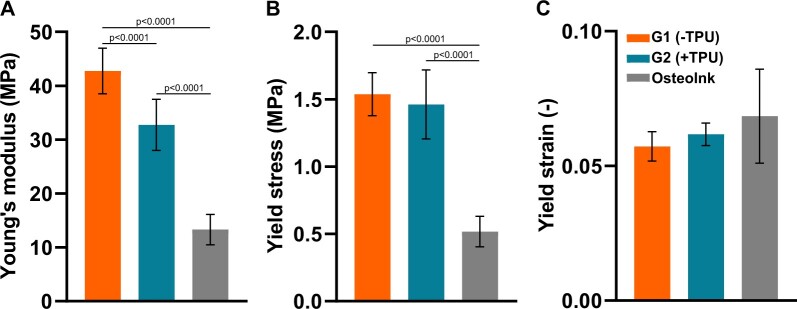
Mechanical properties of 3D-printed scaffolds (Design 1: (G1 and G2: *N* = 14, Osteoink: *N* = 13): (**A**) Young’s modulus, (**B**) yield stress and (**C**) yield strain.

The addition of TPU significantly decreases the Young’s modulus (Figure 7A) of 3D-printed scaffolds from 43 ± 4.2 MPa in G1(−TPU) to 33 ± 4.8 MPa in G2(+TPU) (Design 1) under cyclic mechanical compression, while no difference is measured for the yield stress (Figure 7B) (G1(−TPU): 1.5 ± 0.16 and G2(+TPU): 1.5 ± 0.26 MPa) and yield strain (Figure 7C) (G1(−TPU): 0.06 ± 0.005 and G2(+TPU): 0.06 ± 0.004). Both groups show significantly increased Young’s modulus and yield stress compared to the OsteoInk^®^ (13 ± 2.8 MPa and 0.5 ± 0.1 MPa, respectively), but not for the yield strain (0.07 ± 0.02).

### Scaffolds of both groups resist drilling and screwing

**Figure 8.**
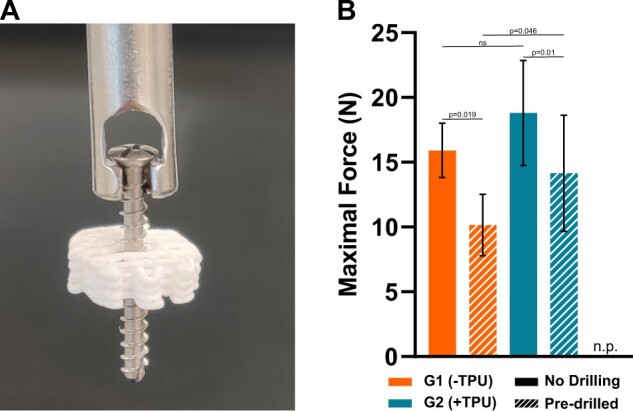
Screw pull-out test: (**A**) representative image of the screw within the 3D-printed scaffold, screw length: 18 mm; (**B**) maximal force with and without pre-drilling (*N* = 12); n.p. = not possible to perform test on OsteoInk, due to scaffold breakage upon drilling or screwing, ns = not significant.

Drilling using a 1.8-mm drill head and screwing using 2 mm medical grade screw does not break or impair the 3D-printed scaffolds (Design 1) (Figure 8A) but does break the OsteoInk^®^ scaffold (Supplementary Video), which therefore cannot be used for the screw pull-out test. To pull out the screw from pre-drilled scaffolds of G1(−TPU) a maximal force of 10 *N* ± 2.4 is required, while a significantly increased force of 14 *N* ± 4.5 is required for G2/+TPU (Figure 8B). The force is significantly decreased compared to the scaffolds in which no pre-drilling was performed for both groups (Figure 8B). The addition of TPU leads to an increased trend of maximal force, but no statistical significance was calculated with G1(−TPU) being at 16 ± 2.1 N and G2(+TPU) being at 19 ± 4.1 N.

### The addition of TPU decreases proliferation of seeded hBM-MSCs after 7 days of culture

**Figure 9.**
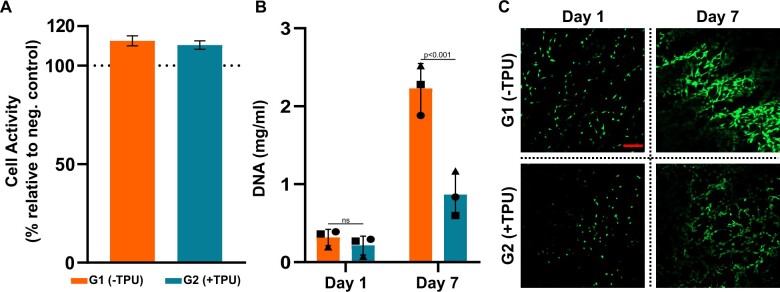
Biological assessment: (**A**) indirect cytotoxicity of L929 mouse fibroblasts activity relative to neg. control on plastic based on cell titer blue^®^ (*N* = 4); (**B**) DNA quantification at day 1 and 7 of three independent donors (*N* = 3: donor A (●), donor B (■) and donor C (▲) of MSCs seeded directly on 3D-printed discs; (**C**) live/dead staining of donor B at day 1 and day 7, scale bar: 200 µm. ns, not significant.

Biocompatibility of 3D-printed scaffolds (Design 1) is investigated via an indirect cytotoxicity test using the L929 fibroblast cell line. Cells are cultured with conditioned media from both scaffold groups and showed metabolic activity greater than 100% when normalized to cells in control medium (Figure 9A).

DNA quantification of hBM-MSCs from three independent donors directly seeded on 3D-printed discs (Design 2) shows proliferation from day 1 to day 7 for both groups. While no difference is measured between the two groups at day 1, a statistically significant difference is observed between G1(−TPU) and G2(+TPU) at day 7 (Figure 9A). The proliferation is confirmed by live/dead images (Figure 9A) in which more live cells are visible on G1(−TPU) compared to G2(+TPU) at day 7. Both groups show no cytotoxicity, while G1(−TPU) favours cell proliferation compared to G2(+TPU).

### β-TCP particles are homogeneously distributed on 3D-printed discs of both groups

**Figure 10.**
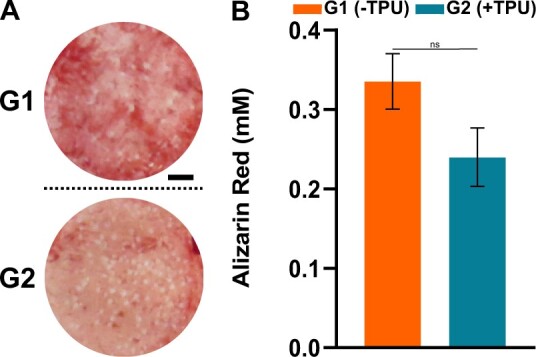
Surface calcium staining of 3D-printed discs with Alizarin red: (**A**) representative images; (**B**) Alizarin red quantification (*N* = 3), scale bar = 2 mm. ns, not significant.

To understand the β-TCP exposure on the scaffolds, 3D-printed discs (Design 2) are stained with Alizarin red. β-TCP particles are homogeneously distributed on the discs for both groups and the addition of TPU leads to a decrease of β-TCP exposure, visible with less red stained surface on G2(+TPU) (Figure 10A). The measured concentration of Alizarin red confirms the images with a trend of higher dye concentration in G1(−TPU) compared to G2(+TPU), but with no statistically significant difference (Figure 10B). These findings are in accordance with the increased ratio of β-TCP within the scaffold in G1(−TPU) compared to G2(+TPU) as a consequence of the TPU addition.

### hBM-MSCs undergo osteogenesis when seeded on 3D-printed discs G1(−TPU)

**Figure 11.**
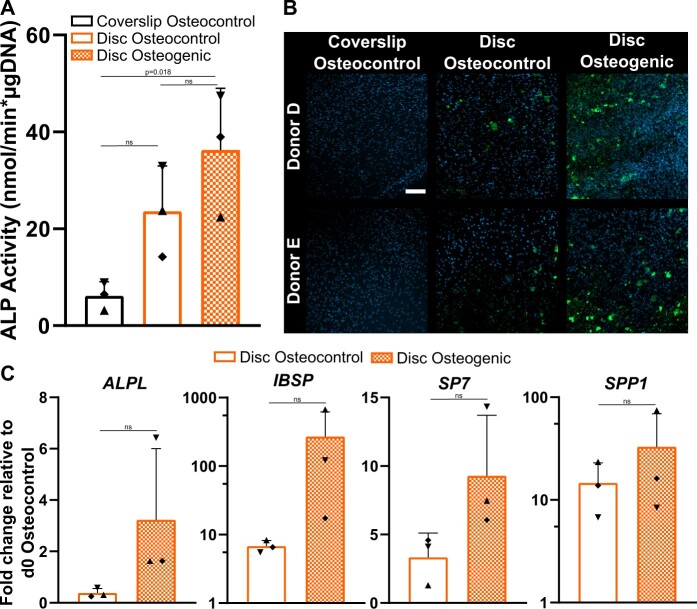
Osteogenic assessment of hBM-MSCs cultured on G1(−TPU) of three independent donors (*N* = 3): (**A**) ALP activity normalized to DNA content at day 14; (**B**) staining of mineral deposition (green) and nucleus (blue) at day 28 of two representative donors, scale bar: 200 µm; (**C**) gene expression of osteogenic markers (*ALPL*, *IBSP*, *SP7* and *SPP1*). Each data point represents each individual donor: donor C (▲), donor D (♦) and donor E (▼). ns, not significant.

In light of the enhanced proliferation profile of G1(−TPU) (Figure 9B and C), this group was selected to carry out the osteogenic assay using hBM-MSCs of three independent donors. Cells are seeded and cultured on either coverslip under osteocontrol condition or on 3D-printed discs (Design 2) under osteocontrol and osteogenic condition for 28 days. ALP activity at day 14, normalized to the DNA content is upregulated in both disc groups compared to the coverslip osteocontrol group, and in the disc osteogenic group compared to the disc osteocontrol group (Figure 11A). Confocal images of the fluorescent-stained mineral deposition at day 28 show visibly increased mineralization in the disc osteogenic group compared to the disc osteocontrol group, while no mineralization is detected in the coverslip osteocontrol group (Figure 11B). Gene expression of hBM-MSCs on 3D-printed discs show an upregulation of *ALPL*, *IBSP*, *SP7* and *SPP1*, all relevant makers for osteogenesis, when cultured under osteogenic conditions compared to control conditions without statistical significance (Figure 11C).

ALP activity and mineral deposition indicates osteogenic differentiation of hBM-MSCs when cultured on G1(−TPU) discs, with enhanced effects under osteogenic culture conditions, especially visible looking at the gene expression of osteo-relevant markers.

### Solvent-based printing can create precise large-scale scaffolds

**Figure 12.**
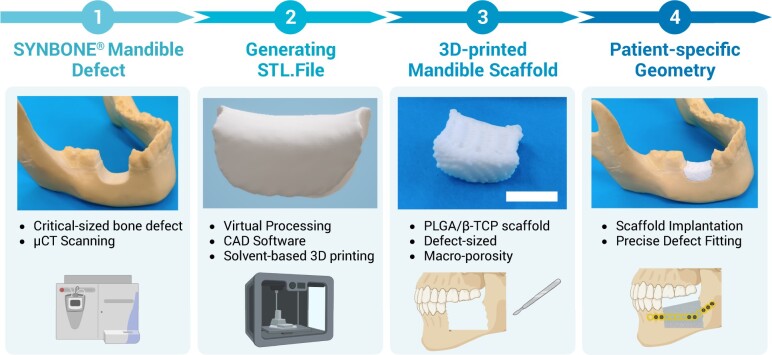
Workflow of the creation of a patient-specific 3D-printed scaffold via (1) µCt scanning of a critical-sized defect, (2) generating and virtual processing the STL. file, (3) solvent-based printing of a defect-sized scaffold based on PLGA/β-TCP (G1(−TPU)) and (4) precise defect implantation of the scaffold, scale bar = 1 cm. Created with BioRender.com.

To further demonstrate the clinical compatibility of this biofabrication tool, a precise and patient-specific mandible defect-sized scaffold based on PLGA/β-TCP is printed using the solvent-based printing approach (Figure 12). The macro-porous 3D-printed scaffold shows precise defect fitting upon implantation in the defect site, a highly desired characteristic for patient’s aesthetics.

## Discussion

The purpose of this study was to develop a 3D-printing fabrication technique to create porous scaffolds for bone tissue engineering. To increase the availability of different bone substitutes and maximize the possibility to eventually replace the current SOC of autologous bone grafting for the repair of bone defects an easy, cheap and versatile biofabrication process is advantageous. By applying a low-temperature solvent-based printing approach, we successfully fabricated a bone substitute with a three-level macro-, micro- and nano-porosity based on a new formulation PLGA/β-TCP ± TPU with adequate fidelity, cytocompatibility and osteogenic potential. To 3D print PLGA/CaP-based materials for the creation of bone substitutes, the heat-melt printing approach has been generally used, which involves the extrusion of blended granules upon pressure [30–38] and has been applied to variety of PLGA combinations with either HAp [39–42] or TCP [43], but it is associated with FDM disadvantages. As an alternative for 3D printing of PLA-based materials the solvent based printing approach can not only create porous surfaces upon solvent removal of 3D-printed scaffolds but also allows for easy loading and mixing of bioactive factors without the need for post-process chemical modification as previously shown [21]. The presented fabrication method demonstrates the modulation of final material properties such as surface roughness and porosity, both highly connected to osteogenesis outcomes [15, 16] via the addition of TPU to the PLGA/β-TCP composite, which has not been reported previously. To avoid the use of toxic organic solvents such as chloroform [21, 44], dichloromethane [39, 45] or dimethyl sulfoxide [46], commonly used for dissolving the polymeric component, we use EC. It has no toxicity, and it is water soluble. EC solidifies at temperatures lower than 37°C, which allows solidification at lower temperatures and for easy removal in a warm water bath without modification of the fabricated water insoluble polymeric scaffold.

Both inks show viscous like behaviour confirmed by rheological characterization and adequate printability for constructs in the size range relevant to large bone defects of ∼3 cm in height, which is according to the human critical-sized defects of 2.5 cm [47]. Furthermore, the comparison to the commercially available OsteoInk^®^ demonstrates the superior printability of both inks.

3D-printed PLGA/β-TCP ± TPU scaffolds retain fidelity after total EC removal confirmed by NMR analysis and take up ca. 55–65% of aqueous liquid compared to their original weights. Biodegradation plays a pivotal role in facilitating optimal bone healing *in vivo.* For scaffolds to be used as bone graft substitutes, their primary function should be to initiate the bone healing process by serving as initial templates. The scaffolds should gradually degrade as new bone forms, without causing toxic side effects such releasing acidic products [48]. However, determining the ideal degradation rate of an implanted scaffolds is challenging and remains to be elucidated. PLGA, β-TCP and TPU are known to be biodegradable [23, 49, 50]. However, observations from *in vitro* degradation measurements showed no degradation of the 3D-printed scaffolds over a 28-day incubation period in PBS. To obtain accurate values on the degradation rate, long-term *in vivo* degradation experiments would be necessary too, a limitation of this study. The biodegradation of polymers such as PLGA has shown to cause acidic alterations in the surrounding environment [51]. This phenomenon was not observed in the case of the scaffolds presented.

The porosity of the scaffold is an important material property for the adhesion of cells through enhanced adsorption of proteins, due to larger surface area [17] and the potential vessel and bone tissue infiltration into the scaffold *in vivo* [15, 16]. We report the presence of the porosity on the scaffolds in three different levels: the macro porosity determined by the printing design, the micro- and nano-porosity created from the EC removal. The macro pore size of the scaffold is strongly related to neovascularization with larger pore sizes being beneficial for desired ingrowth of blood vessels [52]. *In vivo* fibrous tissue ingrowth is decreasing with increasing pore sizes, with 400 µm being the minimum pore size for optimal blood vessel growth [52], which is in accordance with an average macro pore size of 1 mm of the scaffolds of both groups. Furthermore, interconnected macropores promote body fluid circulation and cell migration to the core of the implant [53]. Previous studies report macro porosities of scaffolds created from solvent-based printing between 70% and 80% [21, 54], much higher than obtained in this study at ca. 32%. Increasing the macro porosity can benefit tissue ingrowth, but decreases mechanical properties [55, 56]. It is therefore important to find the balance of an optimal macro pore size without sacrificing mechanical strength of the scaffold. The presence of surface porosity in the micro [16] and nano [57] range is known to be advantageous for cell adhesion and osteogenesis, but the optimal pore size is still unclear. The average surface pore size of G1(−TPU) is measured to be 60 µm, while the addition of TPU increases the average pore size to 130 µm. The decreased surface porosity upon addition of TPU is credited to the decreased number of pores in G2(+TPU) compared to G1(−TPU). Nano mesoporosity reported from literature regarding the SSA is 6 m^2^/g and an average pore size of 22 nm of PCL/HAp/CNT based on N_2_ sorption higher [45] compared to the reported SSA of 1–2.5 m^2^/g and average pore size of 15–19 nm in this study. Controlling the macro porosity of a 3D-printed scaffold is simple thanks to the nature of 3D printing but challenging for the micro and nano range. The differences in the micro and nano range can be related to processing and the choice of material. Process-related changes such as the choice of the solvent can have an effect: porosities can be created via the evaporation of a volatile solvent or water mediated dissolution such as in this study. Adding an additional material has also shown to change the micro porosity when CNT were added to the PCL/HAp scaffold [45] or TPU to PLGA/β-TCP in this study. The presence of porosity in the three level ranges is a relevant factor for enhanced osteogenic potential of a scaffold and therefore should preferably be available when a new bone substitute is created.

The addition of TPU was hypothesized to increase elasticity, but the presented mechanical properties suggest otherwise. The addition caused a significant decrease in Young’s modulus from 43 MPa in G1(−TPU) to 33 MPa G2(+TPU), which are similar values of PLGA/TCP at 46 MPa from literature [43]. To assess the potential clinical translation mechanical properties of the PLGA/β-TCP ± TPU scaffolds are compared to the commercially available OsteoInk^®^, which shows significantly decreased Young’s modulus and yield stress. Natural bone on the other hand has a much higher stiffness compared to the presented scaffolds: trabecular: 10 GPa and cortical: 19 GPa when tested mechanically [58]. The advantage of adding TPU, known for its high ductility properties [59], is shown by the increased resilience against pulling out screws, which would potentially translate in a less likely mechanical failure of the implant. The brittle nature of OsteoInk^®^ is demonstrated when the screw-pull out test was not possible due to the breakage of the scaffolds beforehand, while the PLGA/β-TCP ± TPU scaffolds remain structurally intact. Mechanical resilience of the scaffold is credited to the inclusion of the mechanical stable polymer PLGA and is an important aspect because the breakage of a bone substitute *in vivo* in a load-bearing environment such as the mandible or femur can have detrimental consequences to the patient’s healing outcome. The lack of precise reproducibility of the printing process with resulting high variances might be explained by the mixing mechanism of the components itself or unstable room conditions due to varying temperature and humidity.

The PLGA/β-TCP ± TPU scaffolds show cytocompatibility when tested with two different cellular types: a L929 mouse fibroblast cell line and primary hBM-MSCS. Additionally, hBM-MSCs showed good adhesion and viability over 7 days of culture. The decreased proliferation rate of hBM-MSCs after 7 days of culture on G2(+TPU) can be explained by lower surface porosity values and decreased β-TCP exposure on the surface or due to the polyurethan’s natural anti-fouling properties [60], but not by the surface hydrophilicity, measured by water contact angle experiment (Supplementary Figure S1). OsteoInk^®^ has shown cytocompatibility with MSCs [61] and allows for micro channel formation of vascular endothelial cells [62]. An Osteoink^®^ like CaP cement paste produced from INNOTERE GmbH showed promising osteogenic *in vitro* and *in vivo* results [63, 64]. The used polymers have shown to be advantages for printability and mechanical stability, but are not bioactive, nor are known to have osteoconductive properties, a disadvantage when included in bone substitutes. The scaffold’s functional osteogenic assessment revealed that hBM-MSCs are capable to differentiate toward an osteoblastic phenotype when seeded on the G1(−TPU) disc during a long-term culture of 28 days. Different methodical assays indicate the same trend of hBM-MSC’s osteogenic response to the osteogenic culture condition, shown by the increased ALP protein expression profile, the stained area of hydroxyapatite, as well as the upregulated gene expression of important osteogenic markers such as *ALPL*, *IBSP*, *SP7* and *SPP1*. ALP, encoded by the *ALPL* gene, a well-known maker for bone formation and calcification [65], provides a high phosphate concentration for osteoblasts during bone mineralization [66]. *IBSP* encodes for bone sialoprotein, a late stage osteoblast differentiation marker [67]. *SP7* encodes for the transcription marker Osterix, which activates the osteogenic differentiation of preosteoblasts into mature osteoblasts [68]. SPP1 encodes for Osteopontin, a late marker for osteogenic differentiation and important player in bone metabolism and homeostasis [69]. Statistical analysis does not show significant differences of osteogenically differentiated hBM-MSCs compared to cells cultured under control conditions, due to a limited donor number and high donor variability, a common consequence when primary hBM-MSCs are tested [70, 71]. However, all donors behaved similarly, the only difference being the magnitude of the response. The PLGA/β-TCP scaffold shows osteoconductive results and can be a promising candidate for further *in vivo* studies but requires additional in-depth *in vitro* investigation using more donors and timepoints to elucidate the mechanism behind the osteogenesis of hBM-MSCs seeded on these 3D-printed scaffolds.

A pre-clinical *in vivo* study to investigate the osteogenic potential of this composite will be required before clinical translation can be undertaken. Future work also aims to upgrade the presented scaffold towards a vascularized bone substitute capable of suppling essential nutrients, cells and oxygen, as well as facilitating nutrient exchange, to promote vascularization and innervation upon implantation into a bone defect, both crucial aspects for successful bone repair [72].

## Conclusion

Low-temperature solvent-based 3D printing is a suitable and versatile fabrication technique to create porous and precise scaffolds composed of a new formulation of PLGA/β-TCP ± TPU scaffolds for bone tissue engineering. The presented 3D-printed scaffolds not only show superior mechanical properties compared to a commercially available CaP ink, but also show adequate cytocompatibility and osteoconductive properties. The addition of TPU to PLGA/β-TCP changes material properties such as porosity and roughness, which influences the osteogenic outcome. The fabrication tool also shows printing scaffolds with a patient-specific geometry and relevant size, that fits precisely into the defect site, which drives towards personalized oral and craniomaxillofacial orthopaedics. The future incorporation of vascularized components is of great necessity for the success of such scaffolds. The constant development and continuous increase in the availability of regenerative bone substitutes maximizes the possibility to eventually replace the current SOC of autologous bone grafting. With regards to clinical application, safety and efficacy need to be further tested in a preclinical bone model.

## Supplementary Material

rbad084_Supplementary_Data
