# Supplementary figures and images for: Micro-porous PLGA/β-TCP/TPU scaffolds prepared by solvent-based 3D printing for bone tissue engineering purposes
Source: Regen Biomater. 2023 Sep 14;10:rbad084. doi: 10.1093/rb/rbad084 (PMC10627288; doi:10.1093/rb/rbad084)

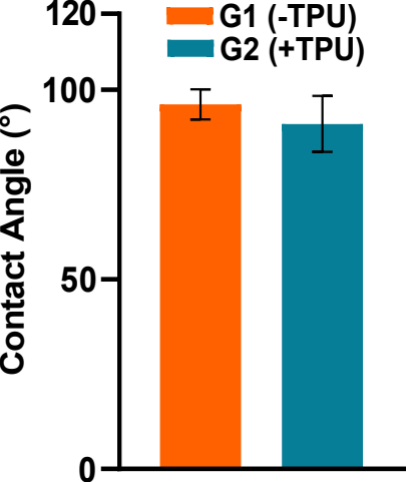

Supplement: rbad084_Supplementary_Data [file rbad084_supplementary_data.pdf]
